# Crystal structure of methyl (*S*)-2-{(*R*)-4-[(*tert*-but­oxy­carbon­yl)amino]-3-oxo-1,2-thia­zolidin-2-yl}-3-methyl­butano­ate: a chemical model for oxidized protein tyrosine phosphatase 1B (PTP1B)

**DOI:** 10.1107/S2056989015010051

**Published:** 2015-06-03

**Authors:** Kasi Viswanatharaju Ruddraraju, Roman Hillebrand, Charles L. Barnes, Kent S. Gates

**Affiliations:** a 125 Chemistry Bldg, University of Missouri Columbia, MO 65211, USA

**Keywords:** crystal structure, iso­thia­zolidine-3-one derivative, oxidized PTP1B, sulfenyl amide, hydrogen bonding

## Abstract

The asymmetric unit of the title compound, C_14_H_24_N_2_O_5_S, contains two independent mol­ecules (*A* and *B*). In each mol­ecule, the iso­thia­zolidin-3-one ring adopts an envelope conformation with the methyl­ene C atom as the flap. In the crystal, the *A* mol­ecules are linked to one another by N—H⋯O hydrogen bonds, forming columns along [010]. The *B* mol­ecules are also linked to one another by N—H⋯O hydrogen bonds, forming columns along the same direction, *i.e.* [010]. Within the individual columns, there are also C—H⋯S and C—H⋯O hydrogen bonds present. The columns of *A* and *B* mol­ecules are linked by C—H⋯O hydrogen bonds, forming sheets parallel to (10-1). The absolute structure was determined by resonant scattering [Flack parameter = 0.00 (3)].

## Abstract 2

The title compound crystallized with two independent mol­ecules (*A* and *B*) in the asymmetric unit. In the crystal, separate chains of *A* and *B* mol­ecules, propagating along the *b*-axis direction, are formed *via* N—H⋯O, C—H⋯S and C—H⋯O hydrogen bonds

## Chemical context   

X-ray crystallographic analyses of the enzyme PTP1B have revealed an unprecedented post-translational modification that may be important in redox regulation of protein function (Zhou *et al.*, 2011 ▸; Salmeen *et al.*, 2003 ▸; van Montfort *et al.*, 2003 ▸; Tanner *et al.*, 2011 ▸; Sivaramakrishnan *et al.*, 2010 ▸). Specifically, oxidation converts the catalytic cysteine in this enzyme to an iso­thia­zolidin-3-one heterocycle that is commonly referred to as a sulfenyl amide residue. As part of early efforts in the area of cephalosporin synthesis, a dipeptide containing a protein sulfenyl amide residue was synthesized (Morin *et al.*, 1973 ▸). However, to the best of our knowledge, there are no examples of low mol­ecular weight sulfenyl amides that have been characterized crystallographically, although structures of related 1,2-benziso­thia­zol-3(2*H*)-ones have been reported (Kim *et al.*, 1996 ▸; Ranganathan *et al.*, 2002 ▸; Wang *et al.*, 2011 ▸). Herein we describe the synthesis and crystal structure of the title compound, a low mol­ecular weight mimic of oxidized PTP1B.
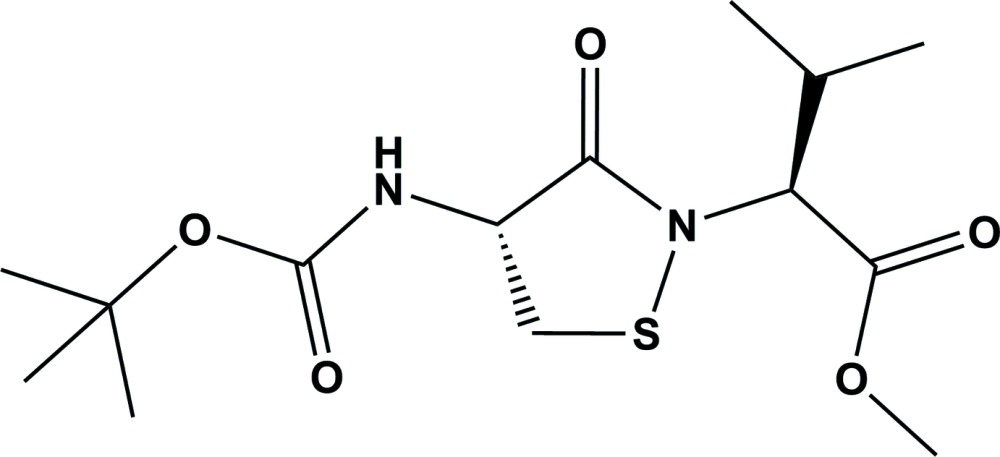


## Structural commentary   

**Figure 1:**
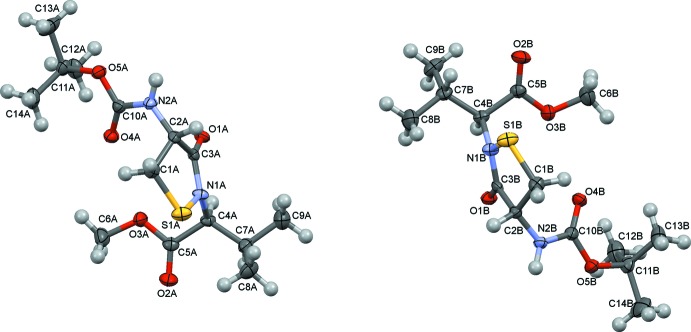
A view of the mol­ecular structure of the two independent mol­ecules (*A* and *B*) of the title compound. Displacement ellipsoids are drawn at the 50% probability level.

The mol­ecular structures of the two independent mol­ecules (*A* and *B*) of the title compound are shown in Fig. 1 ▸. The two mol­ecules differ only in the orientation of the isopropyl group (Fig. 1 ▸). The bond lengths and angles are very similar to those seen in the crystal structures of the oxidized enzyme PTP1B (see: pdb codes 1oem, 1oes, 3sme). In both mol­ecules, the iso­thio­zolidin-3-one ring adopts an envelope conformation with the methyl­ene C atom (C1*A* in mol­ecule *A* and C1*B* in mol­ecule *B*) as the flap, similar to the conformation of oxidized PTP1B (pdb code: 1oem). In previously reported chemical models (1,2-benziso­thia­zole compounds) of PTP1B, the five-membered ring is planar (Kim *et al.*, 1996 ▸; Ranganathan *et al.*, 2002 ▸; Wang *et al.*, 2011 ▸; Sivaramakrishnan *et al.*, 2005 ▸). The S—N bond lengths in the title compound [S1*A*—N1*A* = 1.740 (2) Å and S1*B*—N1*B* = 1.733 (2) Å], are similar to the same bond distance of *ca* 1.71 Å in oxidized PTP1B (pdb code: 1oem).

## Supra­molecular features   

**Figure 2:**
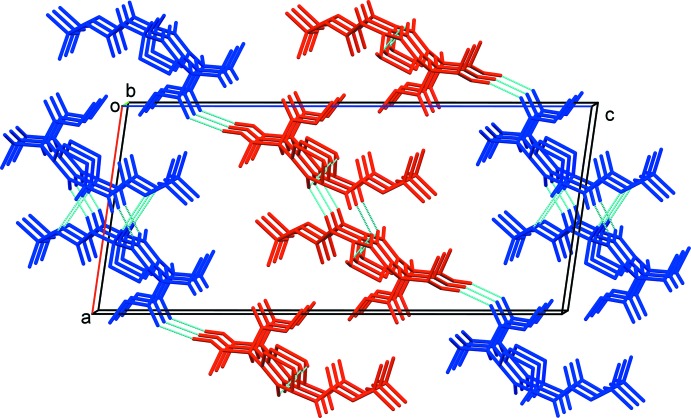
A view along the *b* axis of the crystal packing of the title compound. Hydrogen bonds are shown as dashed lines (see Table 1 ▸ for details; *A* mol­ecules are blue and *B* mol­ecules are red).

In the crystal, N—H⋯O hydrogen-bonding inter­actions give infinite, separate columns of *A* and *B* mol­ecules along the *b*-axis (Table 1 ▸ and Fig. 2 ▸). Within the columns there are C—H⋯S and C—H⋯O hydrogen bonds present (Table 1 ▸). The columns of *A* and *B* mol­ecules are linked by C—H⋯O hydrogen bonds, forming sheets parallel to (10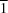); see Fig. 2 ▸.

## Database survey   

A search in the Cambridge Structural Database (Version 5.36; Groom & Allen, 2014 ▸) for the substructure 1,2-benziso­thia­zole-3-one resulted in over twenty hits, which include three structures similar to the title compound: methyl 2-hy­droxy-2-(3-oxobenzo[*d*]iso­thia­zol-2(3*H*)-yl)propano­ate (Ranganathan *et al.*, 2002 ▸), 2-(3-oxobenzo[*d*]iso­thia­zol-2(3*H*)-yl)acetic acid (Wang *et al.*, 2011 ▸) and 2-phenethyl­benzo[*d*]iso­thia­zol-3(2*H*)-one (Kim *et al.*, 1996 ▸). In all three compounds, the five-membered isothizolinone ring is planar. However, the S—N bond lengths are similar to that in the title compound; see *Structural commentary*.

## Synthesis and crystallization   

The title compound was prepared by a modification of a previously published procedure (Shiau *et al.*, 2006 ▸). Pyridine (20 eq) was added to a solution of l-valine ester of *N*,*N*-di-*tert*-butyl­oxycarbonyl-l-cystine (1.0 g, 1.5 mmol) in 50 mL of anhydrous CH_2_Cl_2_. The solution was cooled in a liquid nitro­gen bath, under an N_2_ atmosphere, and stirred for 15 min. Bromine (135 µL, 2.6 mmol) in dry CH_2_Cl_2_ was added dropwise over a period of 30 min. The solution was allowed to warm to 273 K over 1 h, and then CH_2_Cl_2_ was evaporated *in vacuo* using a rotatory evaporator to afford the crude material. Flash chromatography (50% EtOAc/hexa­nes) of the crude material gave the title compound as a white solid (360 mg, 72% yield). Crystals suitable for X-ray diffraction analysis were obtained by slow evaporation of a solution of title compound in DMF.

## Refinement   

Crystal data, data collection and structure refinement details are summarized in Table 2 ▸. The H atoms were included in calculated positions and treated as riding atoms: N—H = 0.88 Å, C—H = 0.98–1.00 Å with *U*_iso_(H) = 1.5*U*_eq_(C) for methyl H atoms and 1.2*U*_eq_(N,C) for other H atoms.

## Supplementary Material

Crystal structure: contains datablock(s) I. DOI: 10.1107/S2056989015010051/su5136sup1.cif

Structure factors: contains datablock(s) I. DOI: 10.1107/S2056989015010051/su5136Isup2.hkl

Supporting information file. DOI: 10.1107/S2056989015010051/su5136Isup3.cml

CCDC reference: 1402668

Additional supporting information:  crystallographic information; 3D view; checkCIF report

## Figures and Tables

**Table 1:** Hydrogen-bond geometry (, )

*D*H*A*	*D*H	H*A*	*D* *A*	*D*H*A*
N2*A*H2*AN*O1*A* ^i^	0.88	2.07	2.925(3)	164
N2*B*H2*BN*O1*B* ^ii^	0.88	2.05	2.921(3)	169
C2*A*H2*A*O5*A* ^i^	1.00	2.57	3.549(3)	167
C1*B*H1*B*2O1*B* ^iii^	0.99	2.56	3.371(4)	139
C4*A*H4*A*S1*A* ^iv^	1.00	2.70	3.526(3)	140
C4*B*H4*B*S1*B* ^iv^	1.00	2.70	3.488(3)	136
C9*B*H9*B*3O2*A* ^v^	0.98	2.52	3.400(4)	149

Symmetry codes: (i) 
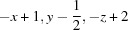
; (ii) 
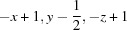
; (iii) 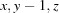; (iv) 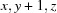; (v) 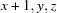.

**Table 2:** Experimental details

Crystal data	
Chemical formula	C_14_H_24_N_2_O_5_S
*M*_r_	332.41
Crystal system, space group	Monoclinic, *P*2_1_
Temperature (K)	173
*a*, *b*, *c* ()	11.509(3), 5.9290(18), 25.751(8)
()	98.307(3)
*V* (^3^)	1738.7(9)
*Z*	4
Radiation type	Mo *K*
(mm^1^)	0.21
Crystal size (mm)	0.50 0.15 0.05

Data collection	
Diffractometer	Bruker APEXII CCD area detector
Absorption correction	Multi-scan (*SADABS*; Bruker, 2008 ▸)
*T*_min_, *T*_max_	0.88, 0.99
No. of measured, independent and observed [*I* > 2(*I*)] reflections	19532, 7699, 6307
*R*_int_	0.026
(sin /)_max_ (^1^)	0.650

Refinement	
*R*[*F* ^2^ > 2(*F* ^2^)], *wR*(*F* ^2^), *S*	0.040, 0.086, 1.05
No. of reflections	7699
No. of parameters	409
No. of restraints	1
H-atom treatment	H-atom parameters constrained
_max_, _min_ (e ^3^)	0.22, 0.25
Absolute structure	Flack *x* determined using 2415 quotients [(*I* ^+^)(*I* )]/[(*I* ^+^)+(*I* )] (Parsons *et al.*, 2013 ▸)
Absolute structure parameter	0.00(3)

Computer programs: *APEX2* and *SAINT* (Bruker, 2008 ▸), *SHELXS2014* (Sheldrick, 2008 ▸), *SHELXL2014* (Sheldrick, 2015 ▸), *Mercury* (Macrae *et al.*, 2008 ▸) and *publCIF* (Westrip, 2010 ▸).

## supplementary crystallographic information

### Crystal data

**Table:**

C_14_H_24_N_2_O_5_S	*F*(000) = 712
*M**_r_* = 332.41	*D*_x_ = 1.270 Mg m^−^^3^
Monoclinic, *P*2_1_	Mo *K*α radiation, λ = 0.71073 Å
Hall symbol: P 2yb	Cell parameters from 7090 reflections
*a* = 11.509 (3) Å	θ = 2.6–22.2°
*b* = 5.9290 (18) Å	µ = 0.21 mm^−^^1^
*c* = 25.751 (8) Å	*T* = 173 K
β = 98.307 (3)°	Needle, colourless
*V* = 1738.7 (9) Å^3^	0.50 × 0.15 × 0.05 mm
*Z* = 4	

### Data collection

**Table:**

Bruker APEXII CCD area-detector diffractometer	7699 independent reflections
Radiation source: fine-focus sealed tube	6307 reflections with *I* > 2σ(*I*)
Graphite monochromator	*R*_int_ = 0.026
ω scans	θ_max_ = 27.5°, θ_min_ = 1.6°
Absorption correction: multi-scan (*SADABS*; Bruker, 2008)	*h* = −14→14
*T*_min_ = 0.88, *T*_max_ = 0.99	*k* = −7→7
19532 measured reflections	*l* = −32→33

### Refinement

**Table:**

Refinement on *F*^2^	Secondary atom site location: difference Fourier map
Least-squares matrix: full	Hydrogen site location: inferred from neighbouring sites
*R*[*F*^2^ > 2σ(*F*^2^)] = 0.040	H-atom parameters constrained
*wR*(*F*^2^) = 0.086	*w* = 1/[σ^2^(*F*_o_^2^) + (0.0332*P*)^2^ + 0.3913*P*] where *P* = (*F*_o_^2^ + 2*F*_c_^2^)/3
*S* = 1.05	(Δ/σ)_max_ = 0.001
7699 reflections	Δρ_max_ = 0.22 e Å^−^^3^
409 parameters	Δρ_min_ = −0.25 e Å^−^^3^
1 restraint	Absolute structure: Flack *x* determined using 2415 quotients [(*I*^+^)-(*I*^-^)]/[(*I*^+^)+(*I*^-^)] (Parsons *et al.*, 2013)
Primary atom site location: structure-invariant direct methods	Absolute structure parameter: 0.00 (3)

### Special details

**Table:**

Geometry. All e.s.d.'s (except the e.s.d. in the dihedral angle between two l.s. planes) are estimated using the full covariance matrix. The cell e.s.d.'s are taken into account individually in the estimation of e.s.d.'s in distances, angles and torsion angles; correlations between e.s.d.'s in cell parameters are only used when they are defined by crystal symmetry. An approximate (isotropic) treatment of cell e.s.d.'s is used for estimating e.s.d.'s involving l.s. planes.

### Fractional atomic coordinates and isotropic or equivalent isotropic displacement parameters (Å^2^)

**Table:**

	*x*	*y*	*z*	*U*_iso_*/*U*_eq_	
S1A	0.17551 (7)	0.37685 (12)	0.89628 (3)	0.0306 (2)	
O1A	0.37713 (16)	0.8791 (4)	0.92762 (7)	0.0258 (5)	
O2A	−0.04290 (17)	0.8246 (4)	0.84881 (8)	0.0391 (6)	
O3A	0.07144 (17)	0.8801 (4)	0.92603 (8)	0.0334 (5)	
O4A	0.25361 (16)	0.8018 (3)	1.02566 (7)	0.0287 (5)	
O5A	0.40692 (16)	0.7475 (3)	1.09140 (7)	0.0277 (5)	
N1A	0.23825 (18)	0.6385 (4)	0.88732 (9)	0.0217 (5)	
N2A	0.40950 (19)	0.5788 (4)	1.01516 (8)	0.0249 (5)	
H2AN	0.4807	0.5362	1.0286	0.030*	
C1A	0.2486 (2)	0.3592 (5)	0.96298 (11)	0.0276 (6)	
H1A1	0.1983	0.4206	0.9877	0.033*	
H1A2	0.2687	0.2009	0.9727	0.033*	
C2A	0.3596 (2)	0.5013 (5)	0.96366 (10)	0.0222 (6)	
H2A	0.4199	0.4062	0.9497	0.027*	
C3A	0.3281 (2)	0.6971 (5)	0.92527 (10)	0.0202 (6)	
C4A	0.1701 (2)	0.8095 (5)	0.85499 (10)	0.0224 (6)	
H4A	0.2129	0.9552	0.8627	0.027*	
C5A	0.0523 (2)	0.8373 (5)	0.87466 (11)	0.0255 (6)	
C6A	−0.0312 (3)	0.8925 (7)	0.95220 (12)	0.0433 (9)	
H6A1	−0.0795	0.7577	0.9439	0.065*	
H6A2	−0.0769	1.0270	0.9403	0.065*	
H6A3	−0.0068	0.9012	0.9902	0.065*	
C7A	0.1596 (3)	0.7759 (5)	0.79545 (11)	0.0300 (7)	
H7A	0.1022	0.8904	0.7788	0.036*	
C8A	0.1133 (3)	0.5449 (6)	0.77637 (12)	0.0399 (8)	
H8A1	0.0416	0.5108	0.7913	0.060*	
H8A2	0.1728	0.4299	0.7876	0.060*	
H8A3	0.0958	0.5455	0.7380	0.060*	
C9A	0.2768 (3)	0.8254 (7)	0.77685 (13)	0.0466 (9)	
H9A1	0.3349	0.7131	0.7916	0.070*	
H9A2	0.3037	0.9763	0.7886	0.070*	
H9A3	0.2672	0.8186	0.7384	0.070*	
C10A	0.3474 (2)	0.7172 (5)	1.04305 (10)	0.0226 (6)	
C11A	0.3593 (3)	0.8922 (5)	1.13002 (11)	0.0299 (7)	
C12A	0.3575 (3)	1.1359 (6)	1.11241 (13)	0.0390 (8)	
H12A	0.3039	1.1522	1.0794	0.058*	
H12B	0.4368	1.1816	1.1071	0.058*	
H12C	0.3308	1.2317	1.1393	0.058*	
C13A	0.4492 (3)	0.8548 (8)	1.17880 (12)	0.0547 (11)	
H13A	0.5272	0.8994	1.1715	0.082*	
H13B	0.4501	0.6949	1.1885	0.082*	
H13C	0.4280	0.9459	1.2078	0.082*	
C14A	0.2390 (3)	0.8122 (6)	1.13960 (13)	0.0423 (9)	
H14A	0.2411	0.6490	1.1458	0.063*	
H14B	0.1811	0.8464	1.1088	0.063*	
H14C	0.2171	0.8894	1.1704	0.063*	
S1B	0.82980 (7)	0.48748 (12)	0.60817 (3)	0.0323 (2)	
O1B	0.61995 (16)	0.9736 (4)	0.57159 (7)	0.0273 (5)	
O2B	1.03914 (18)	0.9218 (4)	0.65232 (9)	0.0431 (7)	
O3B	0.92605 (17)	1.0176 (4)	0.57700 (8)	0.0322 (5)	
O4B	0.74978 (16)	0.8851 (4)	0.47590 (7)	0.0291 (5)	
O5B	0.59660 (16)	0.8252 (4)	0.41038 (7)	0.0289 (5)	
N1B	0.76343 (19)	0.7473 (4)	0.61334 (9)	0.0228 (5)	
N2B	0.5954 (2)	0.6591 (4)	0.48677 (8)	0.0249 (5)	
H2BN	0.5257	0.6095	0.4729	0.030*	
C1B	0.7557 (3)	0.4505 (5)	0.54193 (11)	0.0281 (7)	
H1B1	0.8053	0.5048	0.5161	0.034*	
H1B2	0.7365	0.2897	0.5348	0.034*	
C2B	0.6441 (2)	0.5915 (5)	0.53926 (10)	0.0215 (6)	
H2B	0.5838	0.4986	0.5538	0.026*	
C3B	0.6726 (2)	0.7945 (5)	0.57522 (10)	0.0202 (6)	
C4B	0.8258 (2)	0.9234 (5)	0.64632 (10)	0.0225 (6)	
H4B	0.7808	1.0668	0.6388	0.027*	
C5B	0.9443 (3)	0.9547 (5)	0.62751 (11)	0.0263 (7)	
C6B	1.0263 (3)	1.0160 (8)	0.54915 (13)	0.0472 (9)	
H6B1	1.0737	0.8810	0.5589	0.071*	
H6B2	1.0740	1.1510	0.5584	0.071*	
H6B3	0.9993	1.0148	0.5113	0.071*	
C7B	0.8375 (3)	0.8825 (5)	0.70572 (11)	0.0292 (6)	
H7B	0.8995	0.7658	0.7156	0.035*	
C8B	0.7239 (3)	0.7992 (8)	0.72175 (14)	0.0514 (10)	
H8B1	0.7050	0.6502	0.7063	0.077*	
H8B2	0.6606	0.9050	0.7093	0.077*	
H8B3	0.7323	0.7882	0.7601	0.077*	
C9B	0.8768 (3)	1.1022 (6)	0.73369 (12)	0.0405 (8)	
H9B1	0.8166	1.2180	0.7245	0.061*	
H9B2	0.9508	1.1521	0.7228	0.061*	
H9B3	0.8883	1.0777	0.7717	0.061*	
C10B	0.6558 (2)	0.7978 (5)	0.45889 (10)	0.0242 (6)	
C11B	0.6433 (3)	0.9681 (6)	0.37099 (11)	0.0320 (7)	
C12B	0.6446 (3)	1.2118 (6)	0.38854 (14)	0.0455 (9)	
H12D	0.6719	1.3076	0.3617	0.068*	
H12E	0.6977	1.2282	0.4217	0.068*	
H12F	0.5652	1.2573	0.3936	0.068*	
C13B	0.7631 (3)	0.8901 (7)	0.36143 (14)	0.0492 (9)	
H13D	0.7624	0.7260	0.3567	0.074*	
H13E	0.8214	0.9304	0.3916	0.074*	
H13F	0.7833	0.9630	0.3298	0.074*	
C14B	0.5525 (4)	0.9286 (8)	0.32308 (13)	0.0617 (13)	
H14D	0.5547	0.7702	0.3124	0.093*	
H14E	0.5697	1.0255	0.2943	0.093*	
H14F	0.4742	0.9646	0.3315	0.093*	

### Atomic displacement parameters (Å^2^)

**Table:**

	*U*^11^	*U*^22^	*U*^33^	*U*^12^	*U*^13^	*U*^23^
S1A	0.0326 (5)	0.0190 (4)	0.0366 (4)	−0.0017 (3)	−0.0067 (4)	−0.0018 (3)
O1A	0.0176 (10)	0.0280 (12)	0.0308 (11)	−0.0052 (9)	0.0005 (8)	0.0013 (9)
O2A	0.0231 (11)	0.0551 (16)	0.0367 (12)	0.0073 (10)	−0.0034 (9)	0.0028 (11)
O3A	0.0255 (11)	0.0482 (14)	0.0262 (11)	0.0065 (10)	0.0035 (8)	−0.0077 (10)
O4A	0.0221 (10)	0.0357 (13)	0.0275 (11)	0.0075 (9)	0.0005 (8)	−0.0033 (9)
O5A	0.0258 (11)	0.0327 (12)	0.0236 (10)	0.0037 (9)	−0.0001 (8)	−0.0046 (9)
N1A	0.0168 (11)	0.0180 (12)	0.0288 (13)	0.0022 (9)	−0.0014 (9)	−0.0016 (10)
N2A	0.0201 (12)	0.0323 (14)	0.0214 (12)	0.0059 (10)	−0.0002 (9)	−0.0031 (10)
C1A	0.0310 (15)	0.0246 (15)	0.0273 (15)	−0.0011 (13)	0.0044 (12)	−0.0013 (13)
C2A	0.0183 (13)	0.0266 (15)	0.0215 (14)	0.0064 (12)	0.0025 (10)	−0.0002 (12)
C3A	0.0153 (13)	0.0251 (15)	0.0206 (14)	0.0021 (11)	0.0040 (11)	−0.0017 (11)
C4A	0.0205 (13)	0.0208 (15)	0.0247 (14)	0.0047 (11)	−0.0013 (11)	−0.0015 (11)
C5A	0.0235 (14)	0.0217 (15)	0.0302 (15)	0.0049 (11)	0.0002 (12)	0.0018 (12)
C6A	0.0353 (18)	0.060 (2)	0.0372 (18)	0.0117 (18)	0.0127 (14)	0.0000 (17)
C7A	0.0307 (15)	0.0352 (18)	0.0231 (15)	0.0053 (14)	0.0009 (12)	−0.0019 (13)
C8A	0.049 (2)	0.039 (2)	0.0301 (17)	0.0009 (16)	0.0002 (15)	−0.0087 (14)
C9A	0.044 (2)	0.059 (3)	0.0393 (19)	0.0004 (18)	0.0137 (15)	−0.0010 (17)
C10A	0.0209 (14)	0.0242 (15)	0.0226 (14)	−0.0020 (11)	0.0027 (11)	0.0008 (11)
C11A	0.0335 (16)	0.0327 (18)	0.0242 (15)	−0.0018 (14)	0.0063 (13)	−0.0067 (13)
C12A	0.0441 (19)	0.0284 (18)	0.047 (2)	−0.0075 (15)	0.0164 (16)	−0.0039 (15)
C13A	0.066 (3)	0.066 (3)	0.0271 (18)	0.007 (2)	−0.0105 (17)	−0.0095 (18)
C14A	0.053 (2)	0.042 (2)	0.0364 (18)	−0.0120 (17)	0.0213 (16)	−0.0065 (15)
S1B	0.0358 (5)	0.0192 (4)	0.0375 (5)	0.0025 (3)	−0.0100 (4)	0.0014 (3)
O1B	0.0231 (11)	0.0299 (12)	0.0273 (11)	0.0050 (9)	−0.0020 (8)	−0.0014 (9)
O2B	0.0224 (11)	0.0622 (18)	0.0414 (13)	−0.0038 (11)	−0.0060 (10)	0.0064 (12)
O3B	0.0287 (11)	0.0411 (14)	0.0273 (11)	−0.0026 (10)	0.0054 (9)	0.0025 (10)
O4B	0.0228 (10)	0.0369 (12)	0.0271 (10)	−0.0081 (10)	0.0017 (8)	0.0049 (9)
O5B	0.0277 (11)	0.0373 (13)	0.0204 (10)	−0.0046 (9)	−0.0003 (8)	0.0041 (9)
N1B	0.0228 (12)	0.0168 (12)	0.0269 (12)	−0.0019 (9)	−0.0028 (10)	0.0016 (9)
N2B	0.0213 (12)	0.0314 (14)	0.0208 (12)	−0.0082 (10)	−0.0007 (9)	0.0019 (10)
C1B	0.0312 (16)	0.0242 (16)	0.0285 (16)	0.0035 (12)	0.0034 (13)	0.0009 (12)
C2B	0.0210 (13)	0.0234 (14)	0.0198 (13)	−0.0046 (11)	0.0015 (11)	0.0019 (11)
C3B	0.0132 (12)	0.0253 (15)	0.0225 (14)	−0.0028 (11)	0.0041 (10)	0.0018 (11)
C4B	0.0239 (14)	0.0183 (14)	0.0236 (14)	−0.0009 (11)	−0.0026 (11)	0.0009 (11)
C5B	0.0268 (16)	0.0222 (15)	0.0290 (16)	−0.0040 (12)	0.0011 (12)	−0.0005 (12)
C6B	0.042 (2)	0.061 (3)	0.042 (2)	−0.0102 (19)	0.0164 (16)	−0.0010 (18)
C7B	0.0307 (15)	0.0292 (16)	0.0264 (15)	0.0029 (14)	−0.0009 (12)	0.0044 (13)
C8B	0.050 (2)	0.066 (3)	0.040 (2)	−0.011 (2)	0.0131 (16)	0.0059 (18)
C9B	0.052 (2)	0.040 (2)	0.0262 (16)	0.0019 (16)	−0.0044 (15)	−0.0033 (15)
C10B	0.0219 (14)	0.0279 (16)	0.0228 (14)	0.0028 (12)	0.0032 (11)	0.0009 (12)
C11B	0.0347 (17)	0.0374 (19)	0.0245 (16)	0.0024 (14)	0.0066 (13)	0.0086 (13)
C12B	0.053 (2)	0.037 (2)	0.050 (2)	0.0086 (17)	0.0193 (17)	0.0127 (16)
C13B	0.058 (2)	0.052 (2)	0.043 (2)	0.014 (2)	0.0264 (17)	0.0134 (18)
C14B	0.068 (3)	0.085 (4)	0.0281 (19)	−0.010 (2)	−0.0056 (18)	0.018 (2)

### Geometric parameters (Å, º)

**Table:**

S1A—N1A	1.740 (2)	S1B—N1B	1.733 (2)
S1A—C1A	1.803 (3)	S1B—C1B	1.807 (3)
O1A—C3A	1.215 (3)	O1B—C3B	1.219 (3)
O2A—C5A	1.200 (3)	O2B—C5B	1.199 (3)
O3A—C5A	1.334 (3)	O3B—C5B	1.340 (3)
O3A—C6A	1.444 (4)	O3B—C6B	1.444 (4)
O4A—C10A	1.215 (3)	O4B—C10B	1.222 (3)
O5A—C10A	1.344 (3)	O5B—C10B	1.344 (3)
O5A—C11A	1.477 (3)	O5B—C11B	1.481 (4)
N1A—C3A	1.361 (3)	N1B—C3B	1.356 (3)
N1A—C4A	1.465 (3)	N1B—C4B	1.466 (3)
N2A—C10A	1.360 (4)	N2B—C10B	1.349 (4)
N2A—C2A	1.442 (3)	N2B—C2B	1.443 (3)
N2A—H2AN	0.8800	N2B—H2BN	0.8800
C1A—C2A	1.529 (4)	C1B—C2B	1.526 (4)
C1A—H1A1	0.9900	C1B—H1B1	0.9900
C1A—H1A2	0.9900	C1B—H1B2	0.9900
C2A—C3A	1.534 (4)	C2B—C3B	1.525 (4)
C2A—H2A	1.0000	C2B—H2B	1.0000
C4A—C5A	1.523 (4)	C4B—C5B	1.523 (4)
C4A—C7A	1.533 (4)	C4B—C7B	1.535 (4)
C4A—H4A	1.0000	C4B—H4B	1.0000
C6A—H6A1	0.9800	C6B—H6B1	0.9800
C6A—H6A2	0.9800	C6B—H6B2	0.9800
C6A—H6A3	0.9800	C6B—H6B3	0.9800
C7A—C9A	1.524 (4)	C7B—C8B	1.510 (4)
C7A—C8A	1.525 (5)	C7B—C9B	1.526 (5)
C7A—H7A	1.0000	C7B—H7B	1.0000
C8A—H8A1	0.9800	C8B—H8B1	0.9800
C8A—H8A2	0.9800	C8B—H8B2	0.9800
C8A—H8A3	0.9800	C8B—H8B3	0.9800
C9A—H9A1	0.9800	C9B—H9B1	0.9800
C9A—H9A2	0.9800	C9B—H9B2	0.9800
C9A—H9A3	0.9800	C9B—H9B3	0.9800
C11A—C12A	1.514 (5)	C11B—C13B	1.507 (4)
C11A—C14A	1.518 (4)	C11B—C12B	1.513 (5)
C11A—C13A	1.523 (4)	C11B—C14B	1.516 (4)
C12A—H12A	0.9800	C12B—H12D	0.9800
C12A—H12B	0.9800	C12B—H12E	0.9800
C12A—H12C	0.9800	C12B—H12F	0.9800
C13A—H13A	0.9800	C13B—H13D	0.9800
C13A—H13B	0.9800	C13B—H13E	0.9800
C13A—H13C	0.9800	C13B—H13F	0.9800
C14A—H14A	0.9800	C14B—H14D	0.9800
C14A—H14B	0.9800	C14B—H14E	0.9800
C14A—H14C	0.9800	C14B—H14F	0.9800
			
N1A—S1A—C1A	91.87 (13)	N1B—S1B—C1B	91.57 (12)
C5A—O3A—C6A	116.4 (2)	C5B—O3B—C6B	117.1 (2)
C10A—O5A—C11A	120.8 (2)	C10B—O5B—C11B	121.4 (2)
C3A—N1A—C4A	121.3 (2)	C3B—N1B—C4B	122.3 (2)
C3A—N1A—S1A	114.78 (19)	C3B—N1B—S1B	115.47 (19)
C4A—N1A—S1A	119.59 (17)	C4B—N1B—S1B	119.55 (17)
C10A—N2A—C2A	120.6 (2)	C10B—N2B—C2B	120.5 (2)
C10A—N2A—H2AN	119.7	C10B—N2B—H2BN	119.8
C2A—N2A—H2AN	119.7	C2B—N2B—H2BN	119.8
C2A—C1A—S1A	104.66 (19)	C2B—C1B—S1B	104.80 (19)
C2A—C1A—H1A1	110.8	C2B—C1B—H1B1	110.8
S1A—C1A—H1A1	110.8	S1B—C1B—H1B1	110.8
C2A—C1A—H1A2	110.8	C2B—C1B—H1B2	110.8
S1A—C1A—H1A2	110.8	S1B—C1B—H1B2	110.8
H1A1—C1A—H1A2	108.9	H1B1—C1B—H1B2	108.9
N2A—C2A—C1A	114.0 (2)	N2B—C2B—C3B	111.7 (2)
N2A—C2A—C3A	112.2 (2)	N2B—C2B—C1B	113.9 (2)
C1A—C2A—C3A	106.9 (2)	C3B—C2B—C1B	107.4 (2)
N2A—C2A—H2A	107.9	N2B—C2B—H2B	107.9
C1A—C2A—H2A	107.9	C3B—C2B—H2B	107.9
C3A—C2A—H2A	107.9	C1B—C2B—H2B	107.9
O1A—C3A—N1A	124.2 (2)	O1B—C3B—N1B	123.9 (3)
O1A—C3A—C2A	125.1 (2)	O1B—C3B—C2B	125.4 (2)
N1A—C3A—C2A	110.7 (2)	N1B—C3B—C2B	110.6 (2)
N1A—C4A—C5A	108.3 (2)	N1B—C4B—C5B	106.8 (2)
N1A—C4A—C7A	115.9 (2)	N1B—C4B—C7B	115.4 (2)
C5A—C4A—C7A	113.6 (2)	C5B—C4B—C7B	112.5 (2)
N1A—C4A—H4A	106.1	N1B—C4B—H4B	107.3
C5A—C4A—H4A	106.1	C5B—C4B—H4B	107.3
C7A—C4A—H4A	106.1	C7B—C4B—H4B	107.3
O2A—C5A—O3A	124.7 (3)	O2B—C5B—O3B	124.4 (3)
O2A—C5A—C4A	126.5 (3)	O2B—C5B—C4B	126.8 (3)
O3A—C5A—C4A	108.9 (2)	O3B—C5B—C4B	108.7 (2)
O3A—C6A—H6A1	109.5	O3B—C6B—H6B1	109.5
O3A—C6A—H6A2	109.5	O3B—C6B—H6B2	109.5
H6A1—C6A—H6A2	109.5	H6B1—C6B—H6B2	109.5
O3A—C6A—H6A3	109.5	O3B—C6B—H6B3	109.5
H6A1—C6A—H6A3	109.5	H6B1—C6B—H6B3	109.5
H6A2—C6A—H6A3	109.5	H6B2—C6B—H6B3	109.5
C9A—C7A—C8A	110.8 (3)	C8B—C7B—C9B	111.1 (3)
C9A—C7A—C4A	110.1 (2)	C8B—C7B—C4B	111.7 (2)
C8A—C7A—C4A	114.4 (3)	C9B—C7B—C4B	108.2 (2)
C9A—C7A—H7A	107.1	C8B—C7B—H7B	108.6
C8A—C7A—H7A	107.1	C9B—C7B—H7B	108.6
C4A—C7A—H7A	107.1	C4B—C7B—H7B	108.6
C7A—C8A—H8A1	109.5	C7B—C8B—H8B1	109.5
C7A—C8A—H8A2	109.5	C7B—C8B—H8B2	109.5
H8A1—C8A—H8A2	109.5	H8B1—C8B—H8B2	109.5
C7A—C8A—H8A3	109.5	C7B—C8B—H8B3	109.5
H8A1—C8A—H8A3	109.5	H8B1—C8B—H8B3	109.5
H8A2—C8A—H8A3	109.5	H8B2—C8B—H8B3	109.5
C7A—C9A—H9A1	109.5	C7B—C9B—H9B1	109.5
C7A—C9A—H9A2	109.5	C7B—C9B—H9B2	109.5
H9A1—C9A—H9A2	109.5	H9B1—C9B—H9B2	109.5
C7A—C9A—H9A3	109.5	C7B—C9B—H9B3	109.5
H9A1—C9A—H9A3	109.5	H9B1—C9B—H9B3	109.5
H9A2—C9A—H9A3	109.5	H9B2—C9B—H9B3	109.5
O4A—C10A—O5A	126.4 (3)	O4B—C10B—O5B	125.9 (3)
O4A—C10A—N2A	124.1 (2)	O4B—C10B—N2B	124.4 (2)
O5A—C10A—N2A	109.5 (2)	O5B—C10B—N2B	109.7 (2)
O5A—C11A—C12A	110.1 (2)	O5B—C11B—C13B	111.5 (3)
O5A—C11A—C14A	111.3 (2)	O5B—C11B—C12B	109.3 (3)
C12A—C11A—C14A	112.0 (3)	C13B—C11B—C12B	111.8 (3)
O5A—C11A—C13A	101.4 (3)	O5B—C11B—C14B	101.1 (3)
C12A—C11A—C13A	111.4 (3)	C13B—C11B—C14B	111.1 (3)
C14A—C11A—C13A	110.3 (3)	C12B—C11B—C14B	111.5 (3)
C11A—C12A—H12A	109.5	C11B—C12B—H12D	109.5
C11A—C12A—H12B	109.5	C11B—C12B—H12E	109.5
H12A—C12A—H12B	109.5	H12D—C12B—H12E	109.5
C11A—C12A—H12C	109.5	C11B—C12B—H12F	109.5
H12A—C12A—H12C	109.5	H12D—C12B—H12F	109.5
H12B—C12A—H12C	109.5	H12E—C12B—H12F	109.5
C11A—C13A—H13A	109.5	C11B—C13B—H13D	109.5
C11A—C13A—H13B	109.5	C11B—C13B—H13E	109.5
H13A—C13A—H13B	109.5	H13D—C13B—H13E	109.5
C11A—C13A—H13C	109.5	C11B—C13B—H13F	109.5
H13A—C13A—H13C	109.5	H13D—C13B—H13F	109.5
H13B—C13A—H13C	109.5	H13E—C13B—H13F	109.5
C11A—C14A—H14A	109.5	C11B—C14B—H14D	109.5
C11A—C14A—H14B	109.5	C11B—C14B—H14E	109.5
H14A—C14A—H14B	109.5	H14D—C14B—H14E	109.5
C11A—C14A—H14C	109.5	C11B—C14B—H14F	109.5
H14A—C14A—H14C	109.5	H14D—C14B—H14F	109.5
H14B—C14A—H14C	109.5	H14E—C14B—H14F	109.5
			
C1A—S1A—N1A—C3A	13.0 (2)	C1B—S1B—N1B—C3B	12.9 (2)
C1A—S1A—N1A—C4A	−144.0 (2)	C1B—S1B—N1B—C4B	−148.9 (2)
N1A—S1A—C1A—C2A	−27.2 (2)	N1B—S1B—C1B—C2B	−26.1 (2)
C10A—N2A—C2A—C1A	−61.9 (3)	C10B—N2B—C2B—C3B	57.7 (3)
C10A—N2A—C2A—C3A	59.8 (3)	C10B—N2B—C2B—C1B	−64.2 (3)
S1A—C1A—C2A—N2A	158.7 (2)	S1B—C1B—C2B—N2B	157.0 (2)
S1A—C1A—C2A—C3A	34.2 (3)	S1B—C1B—C2B—C3B	32.7 (3)
C4A—N1A—C3A—O1A	−18.2 (4)	C4B—N1B—C3B—O1B	−15.1 (4)
S1A—N1A—C3A—O1A	−174.7 (2)	S1B—N1B—C3B—O1B	−176.4 (2)
C4A—N1A—C3A—C2A	162.8 (2)	C4B—N1B—C3B—C2B	166.6 (2)
S1A—N1A—C3A—C2A	6.2 (3)	S1B—N1B—C3B—C2B	5.3 (3)
N2A—C2A—C3A—O1A	28.5 (4)	N2B—C2B—C3B—O1B	30.9 (4)
C1A—C2A—C3A—O1A	154.1 (3)	C1B—C2B—C3B—O1B	156.4 (3)
N2A—C2A—C3A—N1A	−152.5 (2)	N2B—C2B—C3B—N1B	−150.9 (2)
C1A—C2A—C3A—N1A	−26.8 (3)	C1B—C2B—C3B—N1B	−25.3 (3)
C3A—N1A—C4A—C5A	−103.6 (3)	C3B—N1B—C4B—C5B	−106.0 (3)
S1A—N1A—C4A—C5A	51.9 (3)	S1B—N1B—C4B—C5B	54.6 (3)
C3A—N1A—C4A—C7A	127.3 (3)	C3B—N1B—C4B—C7B	128.2 (3)
S1A—N1A—C4A—C7A	−77.2 (3)	S1B—N1B—C4B—C7B	−71.3 (3)
C6A—O3A—C5A—O2A	5.4 (5)	C6B—O3B—C5B—O2B	8.6 (5)
C6A—O3A—C5A—C4A	−174.9 (3)	C6B—O3B—C5B—C4B	−169.0 (3)
N1A—C4A—C5A—O2A	−126.7 (3)	N1B—C4B—C5B—O2B	−117.3 (3)
C7A—C4A—C5A—O2A	3.7 (4)	C7B—C4B—C5B—O2B	10.3 (4)
N1A—C4A—C5A—O3A	53.7 (3)	N1B—C4B—C5B—O3B	60.1 (3)
C7A—C4A—C5A—O3A	−176.0 (2)	C7B—C4B—C5B—O3B	−172.3 (2)
N1A—C4A—C7A—C9A	−71.8 (3)	N1B—C4B—C7B—C8B	−44.0 (4)
C5A—C4A—C7A—C9A	161.7 (3)	C5B—C4B—C7B—C8B	−166.8 (3)
N1A—C4A—C7A—C8A	53.7 (3)	N1B—C4B—C7B—C9B	−166.6 (3)
C5A—C4A—C7A—C8A	−72.8 (3)	C5B—C4B—C7B—C9B	70.6 (3)
C11A—O5A—C10A—O4A	1.1 (4)	C11B—O5B—C10B—O4B	1.0 (4)
C11A—O5A—C10A—N2A	179.7 (2)	C11B—O5B—C10B—N2B	−179.4 (2)
C2A—N2A—C10A—O4A	−7.4 (4)	C2B—N2B—C10B—O4B	−4.3 (4)
C2A—N2A—C10A—O5A	173.9 (2)	C2B—N2B—C10B—O5B	176.1 (2)
C10A—O5A—C11A—C12A	−67.5 (3)	C10B—O5B—C11B—C13B	57.3 (4)
C10A—O5A—C11A—C14A	57.3 (3)	C10B—O5B—C11B—C12B	−66.9 (4)
C10A—O5A—C11A—C13A	174.5 (3)	C10B—O5B—C11B—C14B	175.4 (3)

### Hydrogen-bond geometry (Å, º)

**Table:**

*D*—H···*A*	*D*—H	H···*A*	*D*···*A*	*D*—H···*A*
N2*A*—H2*AN*···O1*A*^i^	0.88	2.07	2.925 (3)	164
N2*B*—H2*BN*···O1*B*^ii^	0.88	2.05	2.921 (3)	169
C2*A*—H2*A*···O5*A*^i^	1.00	2.57	3.549 (3)	167
C1*B*—H1*B*2···O1*B*^iii^	0.99	2.56	3.371 (4)	139
C4*A*—H4*A*···S1*A*^iv^	1.00	2.70	3.526 (3)	140
C4*B*—H4*B*···S1*B*^iv^	1.00	2.70	3.488 (3)	136
C9*B*—H9*B*3···O2*A*^v^	0.98	2.52	3.400 (4)	149

Symmetry codes: (i) −*x*+1, *y*−1/2, −*z*+2; (ii) −*x*+1, *y*−1/2, −*z*+1; (iii) *x*, *y*−1, *z*; (iv) *x*, *y*+1, *z*; (v) *x*+1, *y*, *z*.
